# Complex consultations in primary care: a tool for assessing the range of health problems and issues addressed in general practice consultations

**DOI:** 10.1186/1471-2296-15-105

**Published:** 2014-05-27

**Authors:** Sunita Procter, Kate Stewart, David Reeves, Leah Bowen, Sarah Purdy, Matthew Ridd, Chris Salisbury

**Affiliations:** 1 Centre for Academic Primary Care, School of Social and Community Medicine, University of Bristol, Canynge Hall, 39 Whatley Road, Bristol BS8 2PS, UK; 2 University of Nottingham, Medical School at Derby, Royal Derby Hospital, Uttoxeter Road, Derby DE22 3DT, UK; 3 Centre for Primary Care and Centre for Biostatistics, Williamson Building, University of Manchester, Manchester M13 9PL, UK

**Keywords:** Primary health care, Consultation, Clinical coding

## Abstract

**Background:**

There is an increasing recognition that many consultations in general practice involve several problems covering multiple disease domains. However there is a paucity of reliable tools and techniques to understand and quantify this phenomenon. The objective was to develop a tool that can be used to measure the number and type of problems discussed in primary care consultations.

**Methods:**

Thirteen consultations between general practitioners and patients were initially videoed and reviewed to identify the problems and issues discussed. An iterative process involving a panel of clinicians and researchers and repeated cycles of testing and development was used to develop a measurement proforma and coding manual for assessment of video recorded consultations. The inter-rater reliability of this tool was assessed in 60 consultations.

**Results:**

The problems requiring action were usually readily identified. However the different dimensions of the problem and how they were addressed required the identification and definition of ‘issues’. A coding proforma was developed that allowed quantification of the numbers and types of health problems and issues discussed. Ten categories of issues were identified and defined. At the consultation level, inter-rater agreements for the number of problems discussed (within ±1), types of problems and issues were 98.3%, 96.5% and 90% respectively. The tool has subsequently been used to analyse 229 consultations.

**Conclusion:**

The iterative approach to development of the tool reflected the complexity of doctor-patient interactions. A reliable tool has been developed that can be used to analyse the number and range of problems managed in primary care consultations.

## Background

The consultation is described by Pendleton as ‘the central act of medicine’ which ‘deserves to be understood’ [1]. In recent years there has been increasing recognition of the importance in primary health care settings of the fact that many people seeking health care have multiple co-existing problems, or ‘multi-morbidity’ [2-4]. This may impact on a consultation in several ways. For example, the patient may present the general practitioner (GP) with more than one health condition in the same consultation, or they may raise several problems relating to one health condition. The GP may be aware of other co-existing health conditions which influence the management of the presenting problem. In addition, the GP may use the opportunity to discuss or monitor other on-going health conditions.

Although there has been a lot of research on the communication between the GP and patient during a consultation, there have only been a limited number of studies which have tried to quantify, by direct observation, the extent to which multiple problems are discussed in consultations. A better understanding of this may have wide-ranging implications for consultation skills training, consultation length, continuity of health care provider, the skill-mix needed in primary care, and the design of information and record systems. In addition, improved management within consultations may improve patient satisfaction and adherence, which often decline when people have multiple problems and treatments [5].

There has been a long history of research describing the clinical content of general practice, for example national morbidity studies in the UK [6], the BEACH study in Australia [7] and the CONTENT project in Germany [8]. These have assessed the numbers of health problems recorded using existing classification frameworks such as the International Classification of Primary Care (ICPC) Version 2 [9], Read Codes [10], and the International Classification of Diseases (ICD) [11]. However they have all relied on physician self report and research conducted by Rethans et al. [12] showed that medical records did not provide a complete or fully reliable perspective on what takes place within a consultation.

Other studies have not relied on doctor self report and instead used direct observation by researchers or video recording [13,14]. They have sought to understand time spent on different problems within the consultation in order to characterise the patient/clinician interactions with a view to improve the quality of the interaction or for billing purposes. However a common limitation has been the subjectivity of the analysis of the data recorded. Flocke et al. [13] defined 33 descriptive and action categories associated with a health problem as a step to standardising analysis of multiple problems within consultations. Furthermore the use of direct observation by the researcher without video or audio recording of the consultation meant that there was no assessment of inter-rater reliability.

A number of tools are available for analysis of live or recorded consultations, but all are designed to describe the nature and quality of doctor-patient communication rather than the clinical content of the consultation. For example, the Roter Interaction Analysis System (RIAS) [5,15] focuses on understanding the quality of exchange of medical and emotional information. The Davis Observational Coding (DOC) system [16,17] categorises the GPs behaviours during their interaction with the patient into 20 different categories at 15 second intervals. The Measuring Patient Centred Communication scale (MPCC) identifies components of patient centred communication: exploring the patient’s illness experience, understanding the patient as a whole person and finding common ground. Finally, the Verona Patient-Centered Communication Evaluation scale (VR-COPE) [18] builds upon the MPCC [19] and identifies nine communication strategies to accomplish patient centred communication. However none of these lend themselves to understanding or quantifying the number and diversity of problems discussed within a consultation and there is therefore a need to develop a reliable tool.

The “Complex Consultations” project sought to describe the number and types of problem discussed in primary care consultations [20]. As part of the study we developed an analysis tool consisting of a coding proforma and manual to reduce subjectivity in analysing data and we believe this may have use in future studies. The objective of this paper is to describe the development of the analysis tool that could reliably be used to identify and quantify the number and types of health problems managed by GPs in consultations and to assess its reliability, through an evaluation of inter-rater reliability, and also its feasibility.

## Methods

### Source of data

In brief, the “Complex Consultations” project [20] involved the recording of consultations between GPs and their patients during one complete surgery session per GP using a digital video camera. Additionally a review of the computerised medical records made by the GPs was undertaken. A pilot study was conducted with 2 GPs seeing 13 patients and the data collected were used to develop the analysis tool. The main study described elsewhere involved 30 GPs in Bristol, Bath and North Somerset in the south west of England and a total of 229 consultations were videoed between Oct 2010 and June 2011 [20]. The study received ethical approval from South West Central Bristol Local Research Ethics Committee ref 10/H0106/14. Informed consent was obtained from all participants.

### Development of proforma

The starting point for the initial coding proforma was Flocke et al.’s [13] approach. In their methods a problem was operationalised as ‘an issue requiring physician action in the form of a decision, diagnosis, treatment, or monitoring’. In Flocke et al.’s study each problem was coded to one or more of 14 descriptive and 19 action categories and who raised each problem and issue were also recorded. The descriptive categories articulated the nature of the problem being discussed, for example whether it was acute or chronic, administrative, psychosocial or preventive. The action categories captured what the GP did to address the problem including questioning, examination, referral, or arranging laboratory tests.

**Figure 1:**
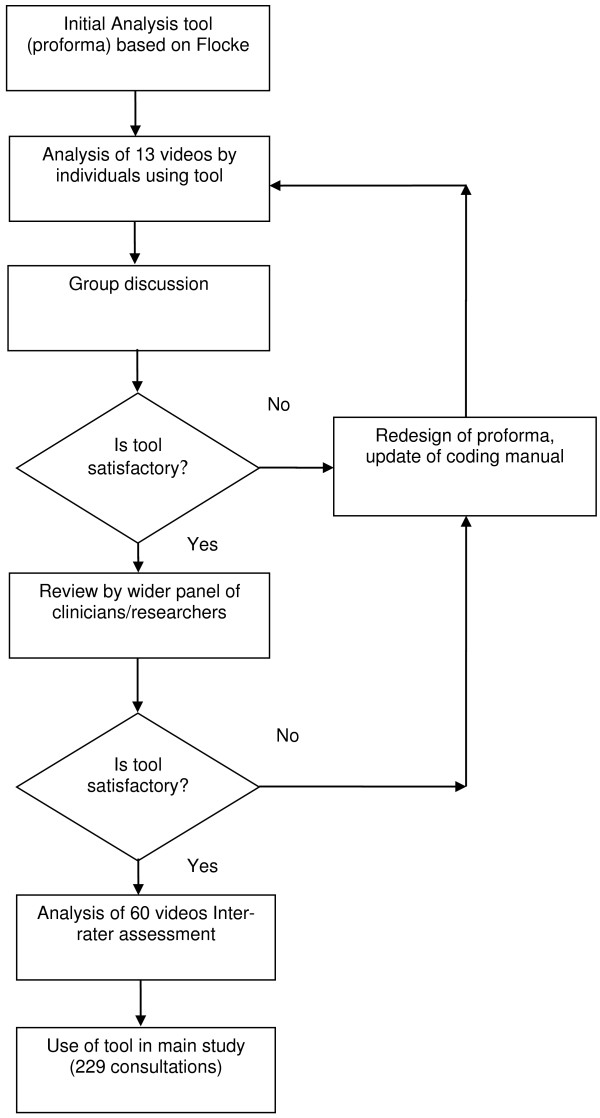
**Diagram depicting development of analysis tool.** Use of tool in main study (229 consultations).

We used the International Classification of Primary Care (ICPC) Version 2 [9] to classify the type of problem in terms of an internationally recognised coding system. The ICPC is particularly suitable because it has chapters (17 in total) for each body system e.g. digestive, circulatory or musculoskeletal, as well as one for common problems encountered in primary care which address multiple chapters (e.g. ‘feeling tired’) plus chapters on psychological problems and social problems. Although we classified each problem using the relevant full ICPC code, for our analysis we amalgamated the problems to chapter level.The development process we followed is illustrated in Figure 1. Five of the authors (SPr, KS, CS, MR, SPu) reviewed the 13 videos from the pilot study using an initial coding proforma individually and then discussed them as a group. During the group discussion it quickly became apparent that there was inconsistency in what each researcher had done and a standardised approach was necessary which led to the development of a coding manual.

This second draft of the proforma and the creation of a coding manual was the start of an iterative process whereby the tool was developed and refined ensuring there was a consistency of understanding and approach to its use. A panel of 8 clinicians and researchers coded and then discussed a sample of 4 video recorded consultations leading to further refinement of the proforma and coding manual.

### Measuring reliability and feasibility

To assess inter-rater reliability two randomly selected videos from each GP who participated in the main study, i.e. 60 consultations, were independently coded by two researchers (SPr and KS). Both researchers have a PhD within the health sciences but are not medically trained. Our final coding tool involved distinguishing between ‘problems’ discussed within each consultation, which were coded within 17 disease areas, and ‘issues’ which were categorised within 10 types. Several problems might fall within the same disease area and each problem might involve more than one issue type. Full details are given in the results. Because the nature of the data was different, inter-rater reliability was computed differently for disease areas and issues than for problems themselves. We computed reliability statistics for:

(i) The number of unique problems recorded. If a rater recorded the same problem code more than once in a consultation it was only counted once. Agreement only concerned the number of problems; the actual problems recorded could differ between raters. We expected the numbers of problems to be in reasonable agreement between raters, but not necessarily to match exactly. We accordingly calculated the% of consultations for which the researchers reported the same number of problems, and the% for which they agreed within plus/minus one problem (e.g. where one researcher coded 3 problems and the other 2 problems). However, high agreement does not necessarily imply an ability to reliably discriminate between consultations, particularly when variation is low [21]. To assess this, we also computed the intraclass correlation coefficient (ICC). Using the methods of Shrout and Fleiss [22] we applied a two-way (consultations by raters) analysis of variance (ANOVA) and then constructed the ICC from the obtained components of variance. We treated the raters as a random effect, for increased generalizability to other raters.

(ii) The issues discussed. For issue types we calculated overall agreement on presence (Positive Agreement; PA) and agreement on absence (Negative Agreement; NA) between the two researchers, as a percentage of all positive (negative) observations. In addition, as a consultation-level measure we computed the number of issues (out of 10) for which both raters agreed on presence or absence, and took the mean across consultations. We also calculated PA and NA for each separate issue.

(iii) Disease areas covered. Overall PA and NA, along with PA and NA for each ICPC disease area, and mean consultation-level agreement, were derived in the same manner as for the issues discussed.

The final version of the proforma was used by one of the researchers (SPr) to analyse the remaining 169 videos of the 229 recorded from the main study. The feasibility of the approach to coding consultations was assessed by how long it took to code each consultation.

## Results

One of the main challenges encountered in developing the coding proforma and manual related to distinguishing between distinct problems and different dimensions or issues relating to each problem. The fluid nature of consultations with cross referencing between different elements and often a non-linear chronology initially made analysis challenging. For example, a patient may have come to discuss their angina (the problem) and the discussion with the GP might have addressed two issues: recent worsening of symptoms (a physical issue) and the need for a medical certificate (an administrative issue). If the patient also mentioned feeling depressed then this would become the focus of discussion of a second problem. After discussion we adopted a clearer description of a problem by adopting a tiered approach in which the ‘problem’ summarises what the GP needs to gather information, make decisions or take actions about (in simple terms, the answer to the question “What is wrong?”), and ‘issues’, which identified the different dimensions or aspects the GP needed to deal with in addressing the problem.

We initially based our description of ‘issues’ on Flocke et al.’s [13] 33 descriptive and action categories, but this proved to be problematic because there were so many categories and they had not been defined. We therefore reduced the number of issues to 10 and developed a definition for each of them. The final version of this is shown in Table 1.

**Table 1:** Issue type definitions

	
**Physical (P)**	Any discussion of or reference to physical symptom, or where the problem is discussed as a physical symptom, disability, or loss of function. (Recording of physical investigations e.g. weight, BP are recorded under ‘**Medicalised health prevention/Maintenance’**)
**Emotional/psychological (EP)**	When the consultation directly addresses psychological or emotional dimensions or consequences of the problem. It is anticipated this will mostly relate to voicing or exploring worries, but is not confined to this. This box does **not** apply if emotional dimensions are just inferred - they have to be addressed.
**Social (S)**	Discussion of the consequences of the problem on the patient’s normal social roles or activities of daily living.
**Administrative (A)**	Dealing with requests for letters and sick notes; making referrals for further consultations; making repeat appointments. Information being sent outwards from the GP for decision making elsewhere.
**Medication related (M)**	Activities relating to any existing medication; any prescription or administration of new medication. Include the direct administration of medication. Includes reviews and re-prescriptions of contraceptive pill.
**Order/refer for tests (OT)**	Issues that raise or resolve the need for tests or investigations to be done beyond the current consultation.
**Discuss test results/treatment (DT)**	Issues that follow up test results, investigations, or treatments (other than medication) that were performed prior to the consultation. Related to information coming inwards from elsewhere, to be acted on by the GP.
**Behavioural health prevention/maintenance (BM)**	As above, but information given or sought relating to *patient actioned* prevention, self-management or risk management issues *behaviours*. Includes discussions of giving up smoking, losing weight, alcohol consumption improving diet, cardiovascular risk assessment. NB if any of these discussions identify a problem, which then leads to a substantial discussion about how to manage this problem (e.g. heavy drinking, obesity), then start a new Problem rather than including this as an issue type.
**Medicalised health prevention/maintenance (MM)**	Information given or sought relating to *GP actioned* patient prevention, self-management or risk management issues. Particularly discussions or investigations which are not relating to a current symptomatic health problem, but are intended to prevent problems in future. Includes taking BP, weighing, discussion of vaccinations, cervical smears and flu jabs. NB if any of these discussions identify a problem, which then leads to a substantial discussion about how to manage this problem (e.g. heavy drinking, obesity), then start a new Problem rather than including this as an issue type.
**3**^**rd **^**party issues (3P)**	Discussion of problems relating to someone **other than the patient**. This does **not** include accounts of others’ comments or views on any of the patient’s problems that are discussed.

From the data extracted from the medical records we decided to add to the proforma whether the problem had been recorded in the notes and if so whether it was just recorded in free text or whether it was also Read coded. Read codes are the classification system used in all GP computer systems in the UK [10]. This enabled us to explore secondary research questions about whether problems discussed in consultations are also recorded in the records. Referring to the records was also useful because in a small number of cases it was clear that a problem was being discussed, but impossible to tell from the videoed conversation the nature of the problem. In such cases our coding rules allowed the researcher to use the medical records to clarify the nature of the problem, and such instances were recorded on the proforma to distinguish them from cases coded independently of the medical records.

**Figure 2:**
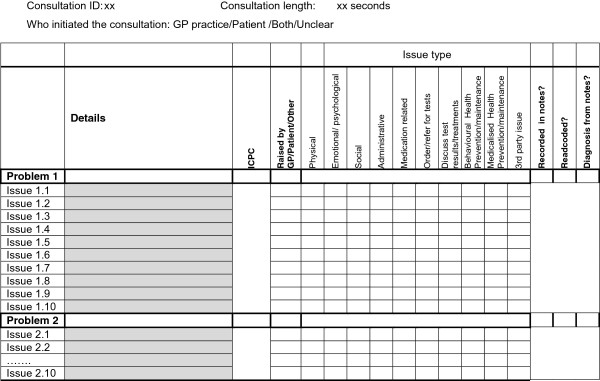
Coding proforma.

The final coding proforma developed is shown in Figure 2. It includes a consultation identification number, and also details of the length of the consultation (derived from the video-recording) and who initiated the consultation (if this was evident from the recording). The ‘problems’ are described briefly in free text using the same words spoken by the GP or patient e.g. back ache to provide the link to the data and later classified using ICPC codes e.g. L02 (back symptom/complaint) for performing the quantitative analysis. There are spaces for up to ten ‘issues’ per problem as this was found to be necessary in the main study. Each problem may include one or more issues, and each issue can be of more than one issue type. The ‘issues’ are named and described in Table 1 and were developed as described earlier. Additional columns were included to indicate whether each of the problems discussed in consultations was recorded in the GP’s notes and whether it was Read coded, and also whether the notes had to be used to retrospectively clarify the nature of the problem.

### Reliability and feasibility

The number of problems recorded per consultation by either researcher varied between 1 and 6, though for the great majority of consultations (50 out of 60) 3 problems at most were recorded (Table 2). The two raters agreed exactly on the number of problems discussed in 48 consultations (80%), and they agreed within a difference of 1 problem in 59 (98.3%). The ICC for the number of problems discussed was 0.93.

**Table 2:** **Cross**-**tabulation of the number of problems per consultation recorded by rater 1 and rater 2**

		**Number of problems recorded by rater 2**					
		**1**	**2**	**3**	**4**	**5**	**Total**
Number of problems recorded by rater 1	1	18	1	0	0	0	19
	2	1	15	3	0	0	19
	3	0	0	10	2	0	12
	4	0	0	2	5	1	8
	5	0	0	0	1	0	1
	6	0	0	0	1	0	1
	Total	19	16	15	9	1	60

For the ICPC disease areas a total of 2040 observations were made (2 raters times 17 disease areas times 60 consultations), of which 252 (12.4%) included a positive identification of a disease area and 1788 (87.6%) did not. The overall PA was 87% and the overall NA 98%. At the consultation level, the mean number of disease areas on which the raters agreed (on either presence or absence) was 16.4 (96.5%) with a range of 13 to 17. Analysis of the individual disease areas indicated that agreement on absence was higher than agreement on presence for all areas (Additional file 1 Table S1).

For issue types 1200 observations were made (2 raters times 10 issues times 60 consultations) of which 557 (46.4%) were a positive identification and 643 (55.6%) were negative. At the consultation level, the mean number of issues on which the raters agreed (on either presence or absence) was 9 (90%) with a range of 6 to 10. The overall PA was 89% and the overall NA 91%. Rates for individual issues are reported in Additional file 2 Table S2.

The researcher who coded the 169 remaining videos from the main study took typically 15 minutes longer than the actual consultation to complete the proforma.

## Discussion

### Summary

The final coding proforma (Figure 2) records the different problems raised, and the different dimensions (‘issues’) of that problem, the ICPC code as well as a number of other data. The inter rater agreement statistics were high for all three measures, indicating excellent agreement about which issues/disease areas were discussed - and also not discussed - in the consultations. The high ICC for number of problems suggests that the tool is also good at discriminating between consultations on this measure. The time taken to code the videos was also acceptable.

### Comparison with existing literature

This tool differs from other consultation analysis tools in that it focuses on understanding the complexity of the consultation rather than the quality of the interaction. Also it is based on direct observation of the consultation using video recordings rather than physician self-report which allows for assessment of inter–rater reliability. Earlier research based on observation of multiple problems in consultations has not used any tool to systematically analyse the consultation and previous studies have not assessed the reliability of the approach used. Flocke et al. [13] defined a range of categories with the data collected by medical students who were present at the consultation and so there was no way of testing inter-rater reliability. Whilst Tai Seale et al. [14] videoed consultations, they focused on identifying the problem (or ‘topic’) and how the conversation flowed between patient and physician to determine the time spent on each topic. The DOC system [16,17,23] categorises the consultation into times for a number of categories, which are similar to the issues identified here, but does not quantify the number of health problems.

### Strengths and limitations

As far as we are aware, this is the first study to develop a tool to quantify the number of health problems discussed in a consultation within the primary care setting. The limitations of the tool are recognised in that a relatively small number of raters and sample size was used to test inter-rater reliability and no additional information on previous or subsequent consultations was collected. Inter-rater reliabilities were good, particularly in the context of such a fluid and complex interaction as a consultation. It is recognised that the two raters involved in the study were not medically trained and may not be representative of ordinary GPs. The study also considered only inter-rater reliability and not other forms. A future study is needed to more comprehensively test the reliability of the tool. Number of problems recorded is a count variable and is skewed towards small values, whereas the ICC computation strictly assumes data is continuous and normally distributed. However, the ICC was very high suggesting good reliability despite this violation. Further research will be needed to provide greater evidence about the validity of the measure. We have some evidence of construct validity, in that using our measurement tool we have shown anticipated relationships with other variables for example the number of problems discussed is positively associated with patient age and with the length of consultations [20]. Establishing criterion validity is more difficult since there is no appropriate gold-standard for comparison. This is the first study to use this measure and we recommend that additional studies replicate the approach in other patient age groups and practice settings.

### Implications for future research and clinical practice

Being able to quantify the complexity of consultations has the potential to inform policy in a number of areas with a view to improving patient outcomes, for example, the length of consultations needed, the influence of patient-practitioner continuity, or the skills needed by primary care practitioners in their interactions with patients. It also makes it possible to compare consultations in different contexts, such as those run by nurses rather than doctors in general practice, or conducted in settings such as walk-in centres. Additionally it could have implications for research which relies on computerised medical notes recorded by GPs. By reviewing the proportion of problems discussed in consultations which are recorded in notes and whether or not they are Read-coded, this would allow determination of whether there is systematic bias in the types of problems recorded and coded. This would build upon research conducted by Rethans et al. [12] and published by us elsewhere [20].

Through the design and use of this tool, and the associated development of our concept of a ‘problem’, we have had the opportunity to reflect on the nature of what is addressed in primary care consultations. The intricate relationships between presenting ‘problems’ as we have conceptualised them, and both discrete and overlapping health conditions are suggestive of the importance and value of a generalist approach to overall health care. This is highlighted by the difficulties encountered during development of the measurement tool when consultations addressed multiple or overlapping conditions and the way GPs’ checked or monitored non-urgent conditions in subtle ways in between exploration of more pressing ‘problems’. It is important when undertaking research relating to the consultation ‘load’ to engage with the topic in a way that captures these subtleties and intricacies, as illustrated by the complexities which arose in the development of this tool.

## Conclusions

We have developed a reliable tool to quantify the number and range of problems encountered in general practice consultations. The process of developing the tool to quantify the content of patient-GP consultations reflected the complexity of the interactions. The subtleties required a methodical and well defined approach to ensure that there was consistency in the information that could be drawn from interactions.

## Abbreviations

GP: General practitioner; ICPC: International classification of primary care; ICD: International classification of diseases; RIAS: Oter interaction analysis system; DOC: Davis observational coding; MPCC: Measuring patient centred communication scale; VR-COPE: Verona patient-centered communication evaluation scale; ICC: Intraclass correlation coefficient; ANOVA: Analysis of variance; PA: Positive agreement; NA: Negative agreement.

## Competing interests

SPr, KS, DR, LB, SPu, MR, CS had financial support from the NIHR School for Primary Care Research for the submitted work. None of the authors have any competing interests.

## Authors’ contributions

SPr collected the data, contributed to the development of the tool, analysed all the data and drafted the manuscript. KS led the design of the study, participated in the tool development and analysed data to ascertain the inter-rater reliability. DR performed the statistical analysis. SPu and MR participated in the tool development. LB participated in the initial tool development. CS conceived of the study, participated in the tool development and helped to draft the manuscript. All authors read and approved the final manuscript.

## Pre-publication history

The pre-publication history for this paper can be accessed here:

http://www.biomedcentral.com/1471-2296/15/105/prepub

## Supplementary Material

Additional file 1: Table S1 Summary of agreement between raters on presence (positive agreement) and absence (negative agreement) of discussion of disease areas in consultations. Click here for file

Additional file 2: Table S2 Summary of agreement between raters on presence (positive agreement) and absence (negative agreement) of discussion of issues in consultations. Click here for file
