# Supplementary material for: Complex consultations in primary care: a tool for assessing the range of health problems and issues addressed in general practice consultations
Source: BMC Fam Pract. 2014 May 27;15:105. doi: 10.1186/1471-2296-15-105 (PMC4046503; doi:10.1186/1471-2296-15-105)
Supplement: Additional file 1: Table S1 — Summary of agreement between raters on presence (positive agreement) and absence (negative agreement) of discussion of disease areas in consultations. [file 1471-2296-15-105-S1.pdf]

Table S1: Summary of agreement between raters on presence (positive agreement) and absence (negative agreement) of discussion of disease areas in consultations

Disease area	ICPC heading	Positive agreement		Negative agreement	
		N of observations[1]	% Agreement	N of observations[2]	% Agreement
General & unspecified	A	38	84%	82	93%
Blood & immune mechanism	B	10	60%	110	96%
Digestive	D	19	95%	101	99%
Eye	F	5	80%	115	99%
Ear	H	4	100%	116	100%
Cardiovascular	K	10	80%	110	98%
Musculoskeletal	L	51	94%	69	96%
Neurological	N	13	77%	107	97%
Psychological	P	12	83%	108	98%
Respiratory	R	20	100%	100	100%
Skin	S	15	67%	105	95%
Endocrine/metabolic & nutritional	T	16	88%	104	98%
Urological	U	5	80%	115	99%
Pregnancy, childbearing	W	9	67%	111	97%
Female genital	X	9	89%	111	99%
Male genital	Y	8	100%	112	100%
Social problems	Z	8	100%	112	100%
All areas		252	87%	1788	98%

[1]Total number of times area coded as present by one rater or the other

[2]Total number of times area coded as *not present* by one rater or the other
