# Supplementary material for: Complex consultations in primary care: a tool for assessing the range of health problems and issues addressed in general practice consultations
Source: BMC Fam Pract. 2014 May 27;15:105. doi: 10.1186/1471-2296-15-105 (PMC4046503; doi:10.1186/1471-2296-15-105)
Supplement: Additional file 2: Table S2 — Summary of agreement between raters on presence (positive agreement) and absence (negative agreement) of discussion of issues in consultations. [file 1471-2296-15-105-S2.docx]

**Table S2: Summary of agreement between raters on presence (positive agreement) and absence (negative agreement) of discussion of issues in consultations**

| Issue | Positive agreement | | Negative agreement | |
| --- | --- | --- | --- | --- |
|  | N of observations^1^ | % Agreement | N of observations^2^ | % Agreement |
| Physical | 117 | 97% | 3 | 0% |
| Emotional/psychological | 31 | 77% | 89 | 92% |
| Social | 60 | 80% | 60 | 80% |
| Administrative | 5 | 40% | 115 | 97% |
| Medication related | 105 | 99% | 15 | 93% |
| Order/refer for tests | 60 | 87% | 60 | 87% |
| Discuss test results/treatment | 69 | 87% | 51 | 82% |
| Behavioural health prevention | 33 | 85% | 87 | 94% |
| Medicalised health prevention | 65 | 86% | 55 | 84% |
| 3^rd^ party issues | 12 | 67% | 108 | 96% |
| All issues | 557 | 89% | 643 | 91% |

^1^Total number of times issue coded as present by one rater or the other

^2^Total number of times issue coded as *not present* by one rater or the other
